# Microstructure abnormalities of the diffusion quantities in children with attention-deficit/hyperactivity disorder: an AFQ and TBSS study

**DOI:** 10.3389/fpsyt.2023.1237113

**Published:** 2023-08-22

**Authors:** Rui Hu, Fan Tan, Wen Chen, Yong Wu, Yuhan Jiang, Wei Du, Yuchen Zuo, Bingbing Gao, Qingwei Song, Yanwei Miao

**Affiliations:** ^1^Department of Radiology, The First Affiliated Hospital of Dalian Medical University, Dalian, China; ^2^Department of Radiology, Taihe Hospital, Hubei University of Medicine, Shiyan, Hubei, China; ^3^Department of Nuclear Medicine, Taihe Hospital, Hubei University of Medicine, Shiyan, Hubei, China; ^4^Department of Paediatrics, Taihe Hospital, Hubei University of Medicine, Shiyan, Hubei, China

**Keywords:** attention-deficit/hyperactivity disorder, diffusion kurtosis imaging, automated fiber quantification, white matter microstructure, right cingulum bundle

## Abstract

**Objective:**

To explore the specific alterations of white matter microstructure in children with attention-deficit/hyperactivity disorder (ADHD) by automated fiber quantification (AFQ) and tract-based spatial statistics (TBSS), and to analyze the correlation between white matter abnormality and impairment of executive function.

**Methods:**

In this prospective study, a total of twenty-seven patients diagnosed with ADHD (20 males, 7 females; mean age of 8.89 ± 1.67 years) and twenty-two healthy control (HC) individuals (11 males, 11 females, mean age of 9.82 ± 2.13 years) were included. All participants were scanned with diffusion kurtosis imaging (DKI) and assessed for executive functions. AFQ and TBSS analysis methods were used to investigate the white matter fiber impairment of ADHD patients, respectively. Axial diffusivity (AD), radial diffusivity (RD), mean diffusivity (MD) and fractional anisotropy (FA) of 17 fiber properties were calculated using the AFQ. The mean kurtosis (MK), axial kurtosis (AK), radial kurtosis (RK), mean diffusivity (MD_DKI_), axial diffusivity (AD_DKI_), radial diffusivity (RD_DKI_) and fractional anisotropy (FA_DKI_) of DKI and AD, RD, MD, and FA of diffusion tensor imaging (DTI) assessed the integrity of the white matter based on TBSS. Partial correlation analyses were conducted to evaluate the correlation between white matter abnormalities and clinical test scores in ADHD while taking age, gender, and education years into account. The analyses were all family-wise error rate (FWE) corrected.

**Results:**

ADHD patients performed worse on the Behavior Rating Inventory of Executive Function (BRIEF) test (*p* < 0.05). Minor variances existed in gender and age between ADHD and HC, but these variances did not yield statistically significant distinctions. There were no significant differences in TBSS for DKI and DTI parameters (*p* > 0.05, TFCE-corrected). Compared to HC volunteers, the mean AD value of right cingulum bundle (CB_R) fiber tract showed a significantly higher level in ADHD patients following the correction of FWE. As a result of the point-wise comparison between groups, significant alterations (FWE correction, *p* < 0.05) were mainly located in AD (nodes 36–38, nodes 83–97) and MD (nodes 92–95) of CB_R. There was no significant correlation between white matter diffusion parameters and clinical test scores in ADHD while taking age, gender, and education years into account.

**Conclusion:**

The AFQ method can detect ADHD white matter abnormalities in a specific location with greater sensitivity, and the CB_R played a critical role. Our findings may be helpful in further studying the relationship between focal white matter abnormalities and ADHD.

## 1. Introduction

The estimated global incidence of attention-deficit/hyperactivity disorder (ADHD) is approximately 6% (1). ADHD is one of the common neurodevelopmental psychiatric disorders in children and adolescents (2). This disorder is characterized by inattention, excessive impulsivity and hyperactivity. The majority of patients who were diagnosed with ADHD in childhood still suffer from persistent, impairing symptoms in adulthood (3).

There is growing evidence that ADHD is associated with abnormal white matter microstructure (4). Following the advances in analytic techniques of diffusion magnetic resonance imaging (MRI), the use of diffusion MRI in ADHD research has contributed significantly to our understanding of the abnormalities in white matter ultrastructure (5).

At present, studies on white matter changes in ADHD are mostly based on diffusion tensor imaging (DTI) (6–8). Several DTI analytical approaches, such as tract-based spatial statistics (TBSS), voxel-based analysis (VBA), and regions of interest (ROI) analysis, have been widely applied to ADHD (9–11). Nevertheless, the inconsistent outcomes of these approaches can be attributed to variations in analytical techniques and the age disparity among subjects, resulting in diminished white matter integrity across diverse regions and extents. TBSS is a skeleton-based approach which can reduce the effects of local misregistration. A TBSS study showed that there was a decrease in FA in the regions of left genu internal capsule and the right forceps major in ADHD children (12). Unlike this, FA reduction was found to be in the cerebellum, anterior thalamic radiation, and corpus callosum genus in VBA studies (13, 14). In the ROI-based method, ADHD children who were not treated exhibited similar FA values to control children (6). There are advantages and drawbacks to each of these DTI analysis techniques.

It is possible that tissue diffusion characteristics vary along each node of the fiber tract, as diseases injury may occur in some local positions of the bundle. Despite the fact that the majority of studies have found that there are white matter injuries in ADHD, it remains unclear whether the specific properties of white matter abnormalities are different along each fiber tract. Thus, the application of a new automated fiber quantification (AFQ) analysis method is particularly important. Using deterministic tractography, AFQ recreates white matter tracts throughout the brain, so more node-detailed information about white matter changes along each fiber tract could be assessed at an individual level (15). Researchers have recently successfully applied AFQ to many cerebral diseases, such as Alzheimer’s disease. (16–18), epilepsy (19), Cushing disease (20), cerebral small vessel disease (4), and end-stage renal disease (21). To our knowledge, there is currently no AFQ research individually specialized for ADHD in children and adolescents.

Furthermore, crossing fibers can be detected much more accurately with diffusion kurtosis imaging (DKI) than with DTI (22–25). A comparative study found that the DKI parameters were more sensitive to detecting white matter abnormalities with coherent and complex arrangements of fibers than DTI (25). While a few DKI studies have been conducted on ADHD in the past (26, 27), comparative studies between DKI and DTI are lacking. Based on the above factors, we have a hypothesis that there are subtle changes in the microstructure of certain local segments of white matter fibers in ADHD patients. In this study, we plan to perform DKI scans on children with ADHD, and compare white matter fiber changes using TBSS (based on DKI and DTI data) and AFQ (based on DTI data) techniques.

## 2. Materials and methods

### 2.1. Participants

The local ethics committee of Taihe Hospital in Shiyan City approved this prospective study. Informed consent forms were signed by all parents of participating children before the study.

Between August 2022 and February 2023, an MRI screening was conducted on a cohort comprising forty-one ADHD patients and thirty-three healthy controls (HC). The healthy controls were recruited through advertising, while the patients with confirmed ADHD were sourced from our outpatient ADHD clinic. A total of fourteen individuals diagnosed with ADHD and eight healthy control volunteers were excluded from the study due to inadequate image quality or noncompliance with the inspection procedures. Three additional volunteers diagnosed with arachnoid cysts were excluded from the HC group. This study finally included twenty-seven patients with ADHD (20 males, 7 females; 8.89 ± 1.67 years) and twenty-two healthy volunteers (11 males, 11 females; 9.82 ± 2.13 years). The clinical diagnostic criteria of ADHD were fulfilled according to the Diagnostic and Statistical Manual of Mental Disorders, 5th edition (DSM-5) and confirmed either by clinical assessment, both the ADHD indices of parent and teacher were ≥ 75th percentile, which was deemed to be positive for ADHD.

All participants were all 6–13 years of age, right-handed, and did not have any contraindications for MRI scanning. Patient exclusion criteria included: (a) psychiatric diseases other than ADHD, such as depression, anxiety, tic disorder, schizophrenia, etc. (b) medical conditions that may affect brain structure or function, such as craniocerebral trauma, intracranial space-occupying lesions, metabolic disease, hereditary diseases, or other disorders of the central nervous system. (c) MRI contraindications. (d) history of regularly consuming drugs. A healthy control group was recruited with similar age, gender, and education level to the ADHD group and excluded the diagnosis of ADHD. Exclusion criteria for HC: (a) psychiatric diseases, such as ADHD, depression, anxiety, tic disorder, schizophrenia, etc. (b) other exclusion criteria were the same as those for ADHD group.

### 2.2. Clinical characteristics

Clinical data including age, gender, and education years were collected from all subjects. The clinical assessments were completed by an associate chief pediatrician who possesses 12 years of professional experience, including Swanson Nolan and pelham-IV rating scale (SNAP-IV) and the Behavior Rating Inventory of Executive Function (BRIEF).

The SNAP-IV evaluation includes an attention defect score and a hyperactivity score. BRIEF containing 86 items was used for children aged 6 and older to determine executive function abilities. There are two components to the questionnaire: a Behavioral Regulation Index (BRI) and a Metacognition Index (MCI). As part of the BRI, the following three clinical scales are described: inhibit, shift, and emotional control. MCI is composed of the initiate, working memory, plan, organization of materials, and monitor. The original scores of each factor were calculated as T scores after age and gender correction.

### 2.3. Magnetic resonance imaging acquisition

MRI examinations of all participants were performed on a 3.0-Tesla MRI scanner (Signa Architect, GE Healthcare, Milwaukee, WI, United States) equipped with a 48-channel phased-array head coil. In order to reduce noise and minimize head movement, earplugs were used, as well as tight foam padding.

DKI data were obtained using a single-shot spin echo planar imaging (EPI) sequence with the following parameters: diffusion encoding was applied in 60 directions with two b values (b = 1,000 and 2000 s/mm^2^) and one non-diffusion weighted image (b = 0 s/mm^2^), flip angle (FA) = 90^o^, repetition time (TR) = 8,819 ms, echo time (TE) = 90.4 ms, field of view (FOV) = 240 mm × 240 mm, matrix = 120 × 120, NEX = 1, voxel size = 2 mm × 2 mm × 2 mm, slice gap = 0 mm, 75 axial slices, the total acquisition time was 9 min 7 s. Anatomical 3D gradient-echo T1-weighted images were obtained using a 3D gradient-echo sequence to obtain a high-resolution volume image (FA = 12^o^, TE = 3.0 ms, TR = 7.4 ms, FOV = 240 mm × 240 mm, matrix = 240 × 240, slice thickness = 1.0 mm without gap, sagittal slices = 166, scan time = 3 min 47 s).

### 2.4. Diffusion kurtosis data and T1 data preprocessing

A visual inspection of all images was conducted to ensure that only images without visible artifacts were included in the analysis. DKI digital imaging and communications in medicine (DICOM) images were converted into NIfTI (.nii) format and automatically generated b-vectors and b-values using the dcm2nii tool in MRIcron.^1^ The Functional MRI of the Brain (FMRIB) Software Library (FSL) was used to correct distortions caused by eddy currents, correct motion artifacts, and strip skulls. DKI parameters including mean kurtosis (MK), axial kurtosis (AK), radial kurtosis (RK), mean diffusivity (MD_DKI_), axial diffusivity (AD_DKI_), radial diffusivity (RD_DKI_), and fractional anisotropy (FA_DKI_) were calculated using the diffusional kurtosis estimator (DKE). Furthermore, images with b = 0 and 1,000 s/mm^2^ were extracted to calculate parameters [mean diffusivity (MD), axial diffusivity (DA), radial diffusivity (DR), and fractional anisotropy (FA)] of the DTI model by using FSL software. The dcm2nii tool in MRIcron was also used to convert 3D T1WI DICOM images into NIfTI format, then the script mrAnatAverageAcpcNifti and dtiMakeDt6FromFsl were used to align AC (anterior commissure) -PC (posterior commissure) and obtain a dt6 MATLAB format file.

### 2.5. TBSS analysis

The diffusion tensor imaging data underwent processing with the Functional MRI of the Brain (FMRIB) Software Library (FSL) version 5.0.9.^2^ To address eddy current-induced distortions and motion artifacts, each diffusion-weighted image was aligned to the non-diffusion weighted volume (b0 image) through affine alignment, employing the FDT-FMRIB’S Diffusion Toolbox 3.0 tool. The BET-Brain Extraction Tool-v2.1, a component of the FSL package, was utilized to extract a brain mask from the eddy corrected image in order to remove the skull and non-brain tissue. A threshold of 0.2 was set for this purpose. Following this, the DTIFIT tool was employed to estimate the diffusion tensor at each voxel, resulting in the creation of FA, MD, and eigenvalue (λ1, λ2, λ3) maps. The mv and fslmaths commands were subsequently used to generate the parameter graphs for AD and RD, respectively, based on these eigenvalues. The TBSS toolbox in FSL was used for voxel-wise statistical analysis of the FA maps. FA images of all subjects were transformed into Montreal Neurological Institute (MNI) space. An image of the mean FA was generated, then the skeletonized image was used to create a white matter tract skeleton, a FA threshold of 0.20 was used for skeletonization to avoid gray matter and cerebrospinal fluid interference, as well as intersubject variability. We projected aligned FA images of all subjects onto the skeleton. We then applied the same FA transformation to MK, AK, RK, MD_DKI_, AD_DKI_, RD_DKI_, FA_DKI_, MD, AD, and RD images for statistical analysis. To assess the differences in voxel-wise FA, MK, AK, RK, MD_DKI_, AD_DKI_, RD_DKI_, FA_DKI_, MD, AD, and RD between the two groups, the individual skeleton images were subjected to a general linear model (GLM) analysis. Age, sex, and years of education were included as covariates in the analysis. Nonparametric permutation-based testing was conducted using the randomize function in FSL.^3^ The results were considered significant at a *p* < 0.05 level after 5,000 permutations, employing permutation-based non-parametric inference. Threshold-free cluster enhancement (TFCE) and family-wise error (FWE) rate correction were applied to account for multiple comparisons.

### 2.6. AFQ analysis

**Figure 1:**
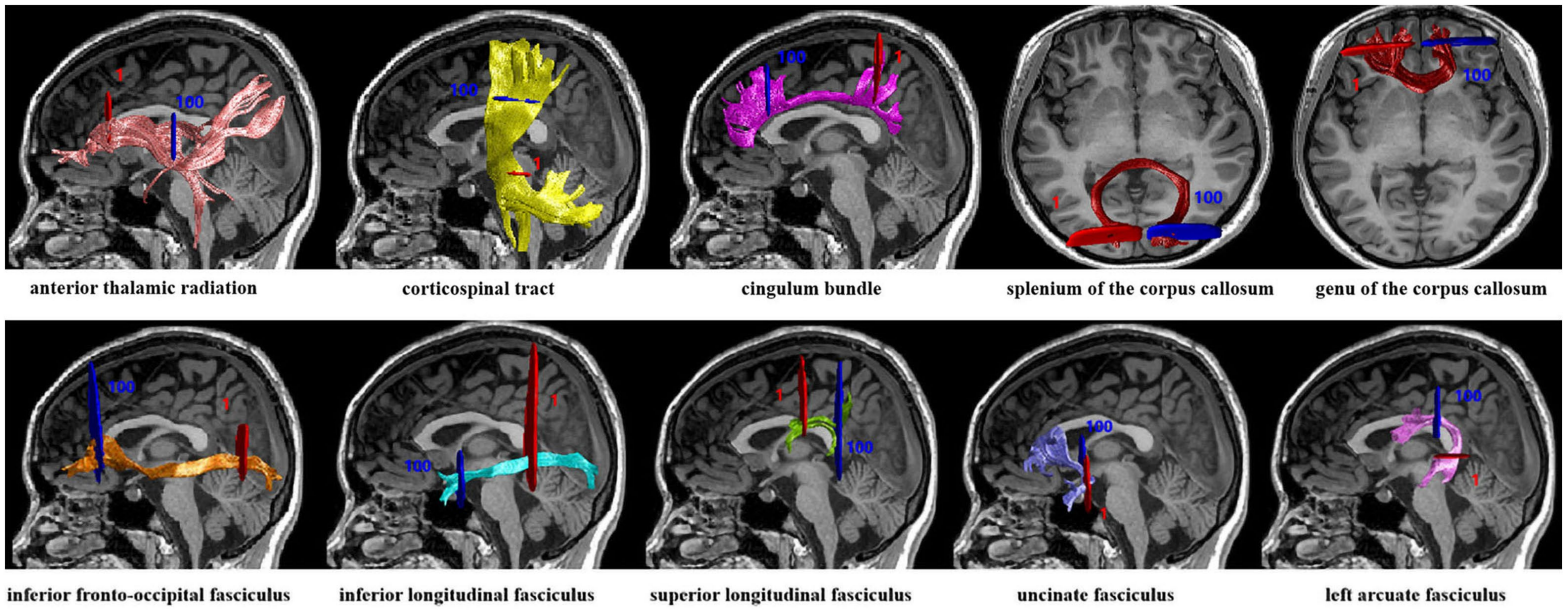
Tracking results from seventeen fiber tract identification. A red region of interest (ROI) represents a starting waypoint, while a blue ROI represents an ending waypoint.

In this study, we identified twenty major fiber tracts of the whole brain and applied AFQ software^4^ to quantify diffusion metrics along these tracts. There are five primary steps in the entire AFQ process: (1) tracking whole-brain fibers with deterministic algorithms. (2) segmentation of fibers based on waypoint region of interest (ROI). (3) refinement of fibers based on probability maps. (4) using the outlier rejection algorithm to clean fiber tracts. (5) measurement of diffusion metrics (FA, MD, AD, RD) at 100 equidistant fiber nodes along each fiber tract. As a result of the threshold setting utilized in fiber tracking, three specific fiber tracts, namely the bilateral cingulum hippocampus (CH_L, CH_R) and right arcuate fasciculus (AF_R), were excluded from further analysis due to their inability to be identified in a significant proportion of participants. This inability to track certain fibers may be attributed to their close proximity to gray matter. For further analysis, we focused on the remaining 17 fiber tracts identified. Following are the 17 identified fiber tracts: bilateral anterior thalamic radiation (ATR_L, ATR_R), corticospinal tract (CST_L, CST_R), cingulum bundle (CB_L, CB_R), splenium of the corpus callosum (CC_S), genu of the corpus callosum (CC_G), inferior fronto-occipital fasciculus (IFOF_L, IFOF_R), inferior longitudinal fasciculus (ILF_L, ILF_R), superior longitudinal fasciculus (SLF_L, SLF_R), uncinate fasciculus (UF_L, UF_R) and left arcuate fasciculus (AF_L). Figure 1 shows the fully identified 17 fiber tracts.

### 2.7. Statistical analysis

The statistical package for social science (SPSS) version 25.0 was used to analyze the clinical data and the mean values of DTI parameters (AFQ) for each fiber tract between ADHD and HC group. Comparing normally and non-normally distributed variables was carried out using independent samples t-tests or Mann–Whitney U-tests. Statistical tests were conducted on enumeration data using Chi-squared or Fisher’s exact tests.

In the statistical analysis conducted using the AFQ method, the average values of DTI parameters (FA, MD, AD, RD) of each fiber tract in the two groups were compared, and multiple comparisons were corrected by FWE correction (*p* < 0.05/68). A point-wise analysis was performed using the “Randomize” command in FSL, as covariates in the GLM model, gender, age, and education years were controlled. Based on 5,000 permutations, a non-parametric permutation-based statistical analysis with FWE (*p* < 0.05) was performed on 1,700 points. As a result, we only reported the results of ≥3 adjacent nodes among those with significant differences. Partial correlation analyses were conducted to determine if the microstructural integrity of white matter correlated with clinical test scores while taking age, gender, and education years into account.

## 3. Results

### 3.1. Demographic and clinical characteristics

Statistical results for demographic and clinical characteristics can be found in Table 1. The groups did not differ significantly in terms of gender, age, and education years (*p* > 0.05). Compared with HC volunteers, ADHD patients performed worse on concentration tests and the BRIEF test and showed significant impairments in inhibit, shift, emotional control, initiate, working memory, plan, organization of materials, and monitor (*p* < 0.05).

**Table 1:** Demographic, clinical data and executive function scores of participants.

Characteristics	ADHD (*n* = 27)	HC (*n* = 22)	Statistical value (*x^2^*/*t*/*z*)	Value of *p*
Gender (M/F)	20/7	11/11	3.023	0.082^c^
Age	8.89 ± 1.67	9.82 ± 2.13	1.712	0.094^a^
Education years	5.85 ± 1.70	6.82 ± 2.13	1.766	0.084^a^
Attention defect score	2.02 ± 0.43	0.76 ± 0.40	−10.497	**<0.001**^a*^
Hyperactivity score	1.58 ± 0.48	0.40 ± 0.33	−9.691	**<0.001**^a*^
Inhibit	53 (49, 62)	42 (40, 49)	−3.923	**<0.001**^b*^
Shift	50 (47, 53)	44 (38,47.25)	−3.076	**0.002**^b*^
Emotional control	51.52 ± 9.15	44.64 ± 7.98	−2.770	**0.008**^a*^
Initiate	56 (43, 61)	43.50 (39.50,51.50)	−2.601	**0.009**^b*^
Working memory	58.78 ± 8.26	47.18 ± 7.52	−5.084	**<0.001**^a*^
Plan	58(52,65)	45(42.75,53.25)	−4.349	**<0.001**^b*^
Organization of materials	55(51,63)	43(37,51.25)	−3.677	**<0.001**^b*^
Monitor	60.52 ± 11.60	46.27 ± 8.73	−4.761	**<0.001**^a*^

^a^Independent samples *t*-test (*t*). ^b^Mann–Whitney U-test (*z*). ^c^Chi-square test (*χ*^2^). *p-values in bold show significant differences (*p < 0.05).

### 3.2. Group differences in tract-based spatial statistics

There were no significant differences in tract-based spatial statistics for MK, AK, RK, MD_DKI_, AD_DKI_, RD_DKI_, FA_DKI_, FA, MD, AD, and RD (*P*-FWE > 0.05, TFCE-corrected).

### 3.3. Group differences in tract level and point-wise level

#### 3.3.1. Mean diffusion differences in tract level

**Figure 2:**
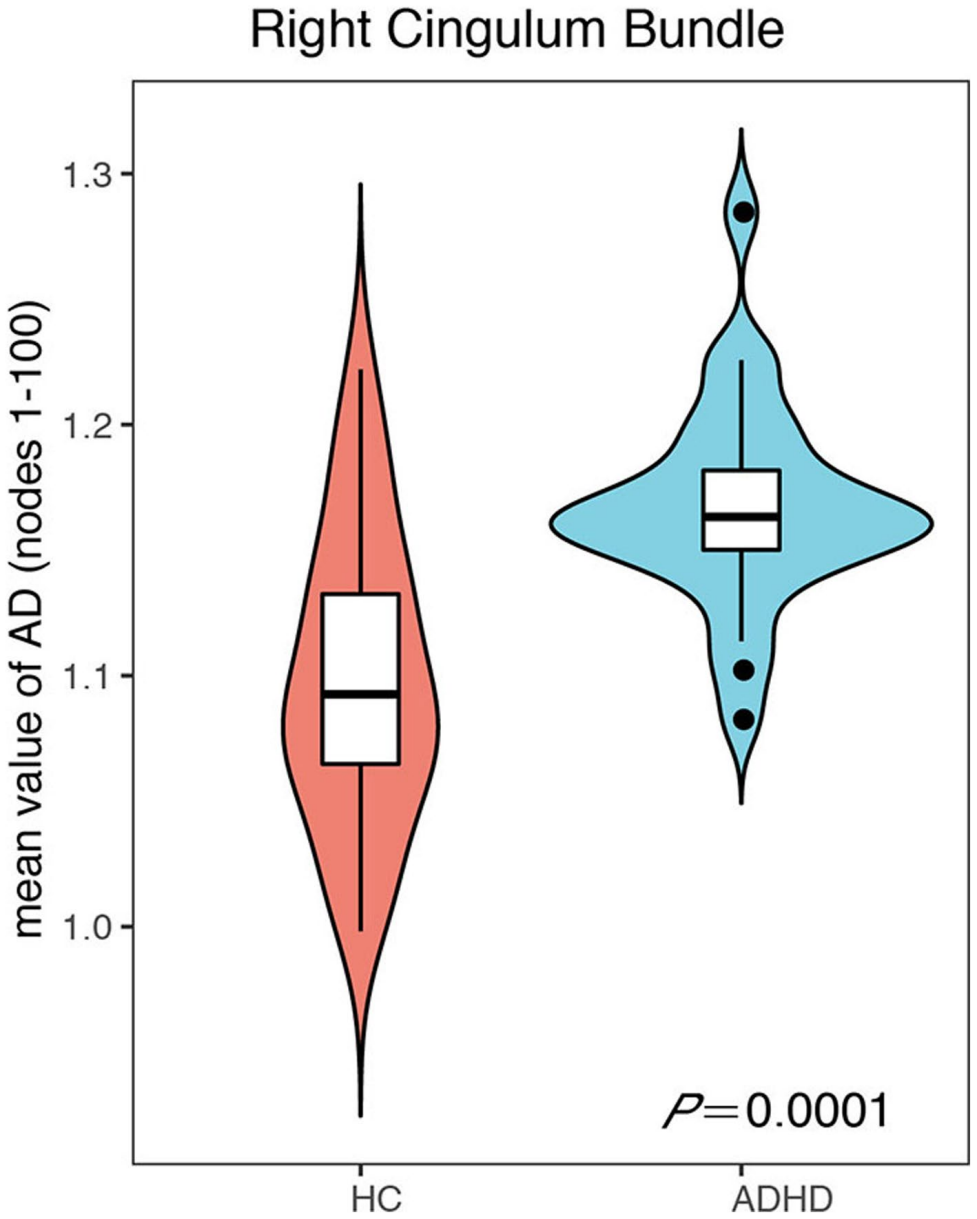
The mean axial diffusivity (AD) value of the right cingulum bundle showed a significant increase in attention-deficit hyperactivity disorder (ADHD) patients following the correction of family-wise error rate.

The mean value of each fiber tracts for FA, MD, AD, and RD were presented in Tables 2–5. Compared to HC, the mean AD value of the CB_R fiber tract showed a significant increase in ADHD patients following the correction of FWE (Table 4; Figure 2).

**Table 2:** Mean FA of each fiber tract comparison between HC and ADHD.

Tract	HC	ADHD	Statistical value (*t/z*)	*p*-value
ATR_L	0.431 (0.412–0.438)	0.432 (0.414–0.452)	−1.126	0.260^b^
ATR_R	0.460 ± 0.029	0.462 ± 0.030	−0.128	0.899^a^
CST_L	0.626 ± 0.020	0.629 ± 0.018	−0.563	0.576^a^
CST_R	0.631 ± 0.019	0.630 ± 0.019	0.127	0.900^a^
CB-L	0.477 ± 0.051	0.498 ± 0.058	−1.289	0.204^a^
CB-R	0.406 ± 0.040	0.442 ± 0.029	−3.596	0.001^a^
CC_S	0.576 (0.520-0.588)	0.570 (0.529–0.604)	−0.342	0.733^b^
CC_G	0.579 ± 0.032	0.590 ± 0.035	−1.131	0.264^a^
IFOF_L	0.457 ± 0.025	0.455 ± 0.027	0.211	0.834^a^
IFOF_R	0.461 ± 0.032	0.469 ± 0.029	−0.925	0.359^a^
ILF_L	0.394 ± 0.032	0.375 ± 0.031	2.098	0.041^a^
ILF_R	0.375 ± 0.024	0.386 ± 0.032	−1.311	0.196^a^
SLF_L	0.406 ± 0.035	0.415 ± 0.049	−0.730	0.469^a^
SLF_R	0.438 ± 0.047	0.445 ± 0.042	−0.552	0.584^a^
UF_L	0.388 ± 0.027	0.394 ± 0.027	−0.739	0.463^a^
UF_R	0.421 ± 0.021	0.425 ± 0.035	−0.525	0.602^a^
AF_L	0.475 ± 0.032	0.480 ± 0.027	−0.526	0.601^a^

^a^Independent samples *t*-test (*t*). ^b^Mann–Whitney U-test (*z*). Bilateral anterior thalamic radiation (ATR_L, ATR_R), bilateral corticospinal tract (CST_L, CST_R), bilateral cingulum bundle (CB_L, CB_R), splenium of the corpus callosum (CC_S), genu of the corpus callosum (CC_G), bilateral inferior fronto-occipital fasciculus (IFOF_L, IFOF_R), bilateral inferior longitudinal fasciculus (ILF_L, ILF_R), bilateral superior longitudinal fasciculus (SLF_L, SLF_R), bilateral uncinate fasciculus (UF_L, UF_R), left arcuate fasciculus (AF_L).

**Table 3:** Mean MD of each fiber tract comparison between HC and ADHD.

Tract	HC	ADHD	Statistical value (*t/z*)	*p*-value
ATR_L	0.741 ± 0.020	0.744 ± 0.024	−0.492	0.625^a^
ATR_R	0.722 ± 0.027	0.720 ± 0.021	0.292	0.771^a^
CST_L	0.758 ± 0.016	0.761 ± 0.018	−0.749	0.457^a^
CST_R	0.746 ± 0.019	0.748 ± 0.017	−0.458	0.649^a^
CB-L	0.731 ± 0.045	0.743 ± 0.030	−1.131	0.264^a^
CB-R	0.743 ± 0.031	0.762 ± 0.028	−2.295	0.026^a^
CC_S	0.932 ± 0.099	0.936 ± 0.072	−0.174	0.862^a^
CC_G	0.753 ± 0.030	0.750 ± 0.039	0.225	0.823^a^
IFOF_L	0.793 ± 0.026	0.797 ± 0.031	−0.459	0.648^a^
IFOF_R	0.779 ± 0.025	0.778 ± 0.020	0.097	0.923^a^
ILF_L	0.837 (0.798–0.853)	0.829 (0.814–0.850)	−0.281	0.778^b^
ILF_R	0.810 (0.791–0.837)	0.815 (0.793–0.834)	−0.422	0.673^b^
SLF_L	0.728 ± 0.036	0.738 ± 0.040	−0.867	0.390^a^
SLF_R	0.738 ± 0.034	0.755 ± 0.035	−1.731	0.090^a^
UF_L	0.833 ± 0.025	0.833 ± 0.032	0.049	0.961^a^
UF_R	0.789 ± 0.031	0.792 ± 0.024	−0.421	0.676^a^
AF_L	0.733 ± 0.033	0.740 ± 0.031	−0.774	0.443^a^

^a^Independent samples *t*-test (*t*). ^b^Mann–Whitney U-test (*z*). Bilateral anterior thalamic radiation (ATR_L, ATR_R), bilateral corticospinal tract (CST_L, CST_R), bilateral cingulum bundle (CB_L, CB_R), splenium of the corpus callosum (CC_S), genu of the corpus callosum (CC_G), bilateral inferior fronto-occipital fasciculus (IFOF_L, IFOF_R), bilateral inferior longitudinal fasciculus (ILF_L, ILF_R), bilateral superior longitudinal fasciculus (SLF_L, SLF_R), bilateral uncinate fasciculus (UF_L, UF_R), left arcuate fasciculus (AF_L).

**Table 4:** Mean AD of each fiber tract comparison between HC and ADHD.

Tract	HC	ADHD	Statistical value (*t/z*)	*p*-value
ATR_L	1.114 ± 0.042	1.129 ± 0.045	−1.249	0.218^a^
ATR_R	1.126 ± 0.042	1.126 ± 0.046	0.012	0.990^a^
CST_L	1.391 ± 0.035	1.404 ± 0.037	−1.277	0.208^a^
CST_R	1.371 (1.359–1.397)	1.386 (1.344–1.418)	−0.543	0.587^b^
CB-L	1.158 ± 0.063	1.202 ± 0.063	−2.425	0.019^a^
CB-R	1.097 ± 0.059	1.166 ± 0.040	−4.662	**<0.001**^a*^
CC_S	1.616 (1.535–1.708)	1.619 (1.594–1.674)	−0.141	0.888^b^
CC_G	1.328 ± 0.050	1.342 ± 0.045	−1.068	0.291^a^
IFOF_L	1.226 ± 0.041	1.230 ± 0.045	−0.297	0.768^a^
IFOF_R	1.200 ± 0.043	1.209 ± 0.044	−0.660	0.513^a^
ILF_L	1.205 ± 0.050	1.199 ± 0.062	0.364	0.718^a^
ILF_R	1.165 (1.134–1.186)	1.175 (1.145–1.227)	−1.347	0.178^b^
SLF_L	1.056 ± 0.053	1.081 ± 0.052	−1.628	0.110^a^
SLF_R	1.106 ± 0.058	1.148 ± 0.063	−2.373	0.022^a^
UF_L	1.212 ± 0.043	1.219 ± 0.050	−0.486	0.629^a^
UF_R	1.184 ± 0.057	1.195 ± 0.047	−0.758	0.452^a^
AF_L	1.139 ± 0.058	1.158 ± 0.042	−1.280	0.207^a^

^a^Independent samples *t*-test (*t*). ^b^Mann–Whitney U-test (*z*). ^*^p-FWE values in bold show significant differences (^*^p < 0.05/68). Bilateral anterior thalamic radiation (ATR_L, ATR_R), bilateral corticospinal tract (CST_L, CST_R), bilateral cingulum bundle (CB_L, CB_R), splenium of the corpus callosum (CC_S), genu of the corpus callosum (CC_G), bilateral inferior fronto-occipital fasciculus (IFOF_L, IFOF_R), bilateral inferior longitudinal fasciculus (ILF_L, ILF_R), bilateral superior longitudinal fasciculus (SLF_L, SLF_R), bilateral uncinate fasciculus (UF_L, UF_R) and left arcuate fasciculus (AF_L).

**Table 5:** Mean RD of each fiber tract comparison between HC and ADHD.

Tract	HC	ADHD	Statistical value (*t/z*)	*p*-value
ATR_L	0.554 ± 0.018	0.551 ± 0.026	0.447	0.657^a^
ATR_R	0.520 ± 0.030	0.517 ± 0.022	0.397	0.693^a^
CST_L	0.441 ± 0.019	0.440 ± 0.018	0.179	0.859^a^
CST_R	0.430 ± 0.020	0.431 ± 0.017	−0.194	0.847^a^
CB-L	0.521 (0.473–0.557)	0.506 (0.473–0.537)	−0.281	0.778^b^
CB-R	0.565 ± 0.035	0.560 ± 0.033	0.518	0.607^a^
CC_S	0.598 ± 0.094	0.593 ± 0.083	0.215	0.830^a^
CC_G	0.465 ± 0.035	0.454 ± 0.046	0.889	0.379^a^
IFOF_L	0.577 ± 0.029	0.581 ± 0.034	−0.426	0.672^a^
IFOF_R	0.568 ± 0.032	0.563 ± 0.026	0.603	0.550^a^
ILF_L	0.645 (0.610–0.667)	0.652 (0.634–0.661)	−0.965	0.335^b^
ILF_R	0.642 (0.607–0.663)	0.634 (0.614–0.659)	−0.020	0.984^b^
SLF_L	0.564 ± 0.037	0.566 ± 0.049	−0.160	0.873^a^
SLF_R	0.554 ± 0.047	0.559 ± 0.041	−0.415	0.680^a^
UF_L	0.647 (0.637–0.665)	0.641 (0.614–0.661)	−0.945	0.345^b^
UF_R	0.591 ± 0.024	0.590 ± 0.030	0.092	0.927^a^
AF_L	0.523 (0.506–0.558)	0.529 (0.507–0.546)	−0.141	0.888^b^

^a^Independent samples *t*-test (*t*). ^b^Mann–Whitney U-test (*z*). Bilateral anterior thalamic radiation (ATR_L, ATR_R), bilateral corticospinal tract (CST_L, CST_R), bilateral cingulum bundle (CB_L, CB_R), splenium of the corpus callosum (CC_S), genu of the corpus callosum (CC_G), bilateral inferior fronto-occipital fasciculus (IFOF_L, IFOF_R), bilateral inferior longitudinal fasciculus (ILF_L, ILF_R), bilateral superior longitudinal fasciculus (SLF_L, SLF_R), bilateral uncinate fasciculus (UF_L, UF_R) and left arcuate fasciculus (AF_L).

The mean AD values for the 16 remaining fiber tracts, as well as the mean FA, MD, and RD values for the 17 fiber tracts, all showed no significant differences after FWE correction (Tables 2–5).

#### 3.3.2. Mean diffusion differences in point-wise level

**Figure 3:**
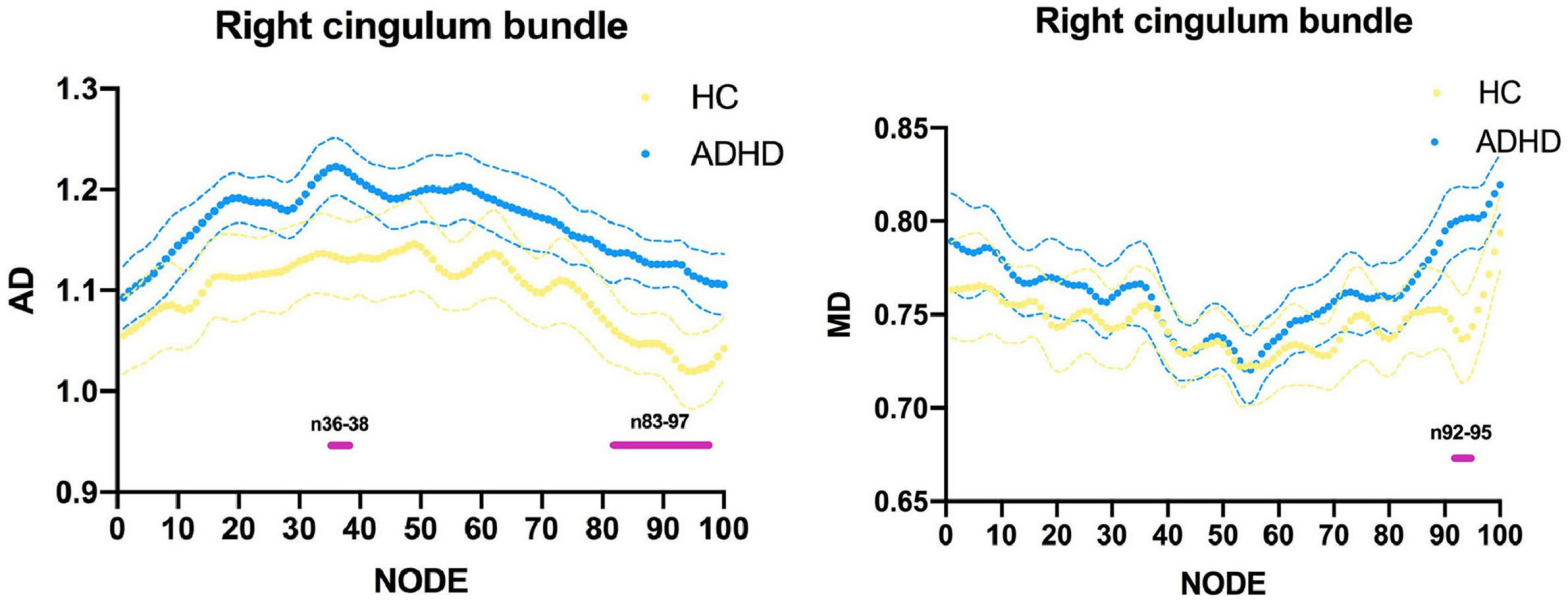
Line chart of the right cingulum bundle with significantly altered in point-wise comparisons of axial diffusivity (AD) and mean diffusivity (MD) values between attention-deficit hyperactivity disorder (ADHD) and healthy control (HC; *p* < 0.05, family-wise error rate correction). ADHD is represented by the blue line, while HC is represented by the yellow line; (the solid lines represent the means and the dotted lines represent the 95% confidence intervals). At the bottom of the graph are the red bars that represent fiber segments with significant differences between groups.

**Figure 4:**
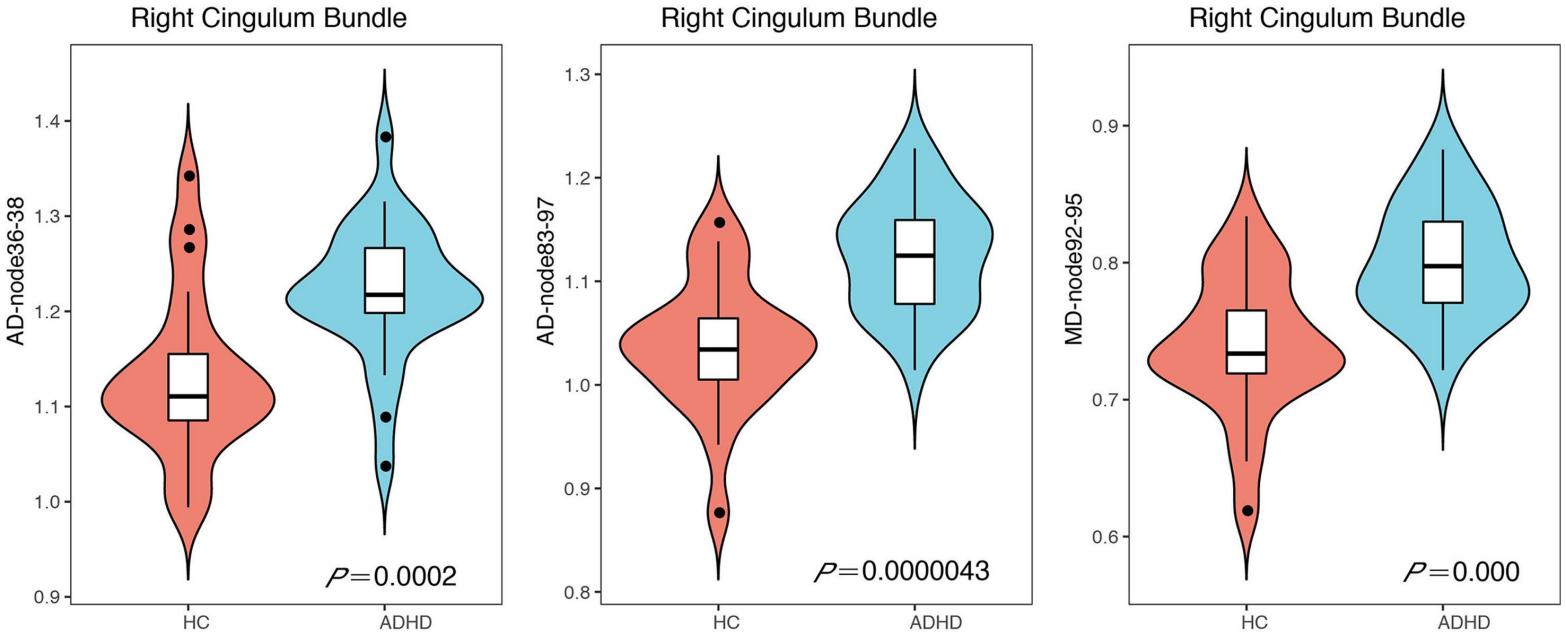
Violin chart showing the Significant results of the point-wise comparison between attention-deficit hyperactivity disorder (ADHD) and healthy control (HC) groups. Significant alterations (family-wise error rate correction, *p* < 0.05) were mainly located in axial diffusivity (AD; nodes 36–38, nodes 83–97) and mean diffusivity (MD; nodes 92–95) of right cingulum bundle.

In order to show detailed diffusion properties of tracts, pointwise comparisons between ADHD patients and HC volunteers were quantified based on AFQ software, white matter disruption is found in specific parts along the white matter tract in ADHD patients. As a result of the point-wise comparison between groups, significant alterations (FWE correction, *p* < 0.05) were mainly located in AD (nodes 36–38, nodes 83–97) and MD (nodes 92–95) of CB_R, details about the significant differences are shown in Figures 3, 4.

### 3.4. Correlation analysis

For the altered AD (nodes 36–38, nodes 83–97) and MD (nodes 92–95) of CB_R, there was no significant correlation between white matter diffusion parameters and clinical test scores in ADHD while taking age, gender, and education years into account.

## 4. Discussion

ADHD patients exhibit specific subcortical dysfunction, with prevailing belief that subcortical abnormalities play a role in the onset of ADHD, while cortical plasticity influences the diverse clinical trajectory of the disorder (28). The thalamus, as the largest subcortical structure in the brain, assumes significance as a crucial element within the cortico striato thalamo cortical circuit (29, 30). Dysfunction within this circuit has been recognized as a significant neural correlate of ADHD (31). Additionally, it is worth noting that the ventral and intralaminar thalamus have been identified as potential components of the somatomotor network (32, 33), and they exhibit connectivity with the anterior midcingulate cortex (34, 35). It is plausible that dysfunctions within these interconnected circuits or subcortical structures might play a role in the manifestation of clinical symptoms associated with attention deficit hyperactivity disorder (ADHD). Additional investigation into ADHD could contribute to a comprehensive comprehension of the disorder. This study exclusively focused on the alterations observed in diffusion imaging, thus necessitating future extensive examinations of ADHD from a multimodal standpoint (36–38).

This study examines the precise modifications in white matter microstructure in children with ADHD through the utilization of AFQ and TBSS. While previous neuroimaging investigations of ADHD have evaluated the integrity of the white matter, the TBSS study outcomes based on voxels did not survive following multiple comparison corrections in this particular study of children. One plausible explanation is that TBSS may be better suited for detecting significant white matter abnormalities (39). For instance, it exhibits sensitivity towards long-term exposure-induced structural alterations (40) or structural changes linked to severe injuries like Traumatic Brain Injury (41). However, the focus of the research cohort pertained predominantly to children who had recently received a diagnosis of ADHD. These individuals are of a tender age and presently in the nascent phases of the disorder, potentially exhibiting alterations solely at a microscopic scale in the white matter fiber bundles. Another possible explanation for the observed outcome could be attributed to the limited scope of the investigation. However, the AFQ analysis revealed the presence of abnormalities in the CB_R, which were more effectively detected by the AFQ method as compared to TBSS. The AD and MD values of CB_R exhibited significant increases at the node-wise level, while only AD values of CB_R showed a significant increase in the entire fiber tract, as per the AFQ study. These findings suggest that AFQ may serve as a complementary method to TBSS, offering a novel approach and providing new insights into ADHD. Notably, node-wise analysis is crucial as it can reveal local anomalies that may be obscured by the whole. Previous research has demonstrated an increase in AD in the right cingulum bundle (42). Our study similarly found a significant increase in the mean AD value of the CB_R fiber tract in individuals with ADHD. AD reflects diffusion coefficients parallel to fiber orientation and is known to be sensitive to axonal damage (43). Therefore, our findings suggest that the observed microstructure changes in the CB_R of individuals with ADHD may be primarily attributed to axonal damage.

The cingulum bundle is a white matter pathway that extends longitudinally from the anterior cingulate cortex (ACC) to the posterior cingulate cortex (PCC), linking the components of the default mode network (DMN) and providing important limbic connections, including those to supplementary motor and primary motor regions (44). This large-scale white matter system (45) is responsible for processing attention and controlling executive functions (46). An amygdala, hippocampus, and insular cortex receive projections from it (47).

There exists a substantial body of evidence indicating that ADHD is linked to deficiencies in various neurocognitive domains. Neuropsychological theories posit that ADHD is chiefly marked by executive dysfunction (48) and anomalous function of the default mode network (DMN) (49). The cingulum bundle is implicated in both executive dysfunction and the DMN theory of ADHD, as supported by these two theories. First, previous studies have shown that executive function processes play a critical role in ADHD (48, 50–52). In executive functioning, the cingulum bundle plays a key role. The cingulum bundle is strongly connected to the cingulate gyrus, which is associated with executive function (53, 54). Second, DMN nodes are connected by various white matter tracts, including anterior and posterior cingulum bundles. Previous studies have demonstrated a correlation between reduced functional connectivity of the cingulum bundle and ADHD. Specifically, researchers using rs-fMRI found that functional connectivity in the DMN and between the dorsal anterior cingulate and posterior region of the DMN was decreased in ADHD adults (55). Additionally, a reduction in brain connectivity has been observed in the anterior cingulate and temporal regions of the brain, which are associated with inattention (56).

At a point-wise level, the AFQ technique enables the measurement of diffusion at 100 equidistant nodes along the fiber tract. In contrast to prior methodologies, this approach affords a more precise analysis of tracts, yielding more accurate identification of damage segments and greater sensitivity to abnormalities in other indicators. The present investigation revealed significant alterations primarily in the AD (nodes 36–38, nodes 83–97) and MD (nodes 92–95) of CB_R. The findings of our study suggest that ADHD patients exhibit injury predominantly in the anterior and posterior regions of the CB_R fiber tract, which is manifested by elevated AD and MD values. Prior research has demonstrated that changes in AD and MD may be indicative of axonal damage (57) and myelin degradation (58). An elevated AD in the CC_R is significantly associated with a decrease in neuronal branching (59). Additionally, the MD metric reflects the diffusion rates of water throughout the brain, and an increase in MD values is indicative of a loss of axons, dendrites, and neuronal cell bodies, ultimately leading to structural alterations at the macroscopic level (60). There is evidence that a reduction in axonal density is linked to heightened MD values (61). Collectively, these findings suggest that the observed microstructural changes in the CB_R are primarily attributed to damage to myelin and axons. Previous research has indicated that the anterior portion of the cingulum bundles plays a crucial role in facilitating communication between limbic regions (62). Furthermore, lesions in this area have been shown to disrupt performance on the 5-Choice Serial Reaction Time Task, which is closely linked to cholinergic activity (63). Given that cholinergic activity is known to be associated with attention, these findings suggest that the anterior cingulum bundles may be involved in attentional processes. Additionally, a recent study has suggested that ADHD may be associated with alterations in the functional connectivity of the right posterior cingulate (64), which may indicate damage to the white matter microstructure of ADHD patients. Drawing upon extant research findings, our investigation uncovered significant alterations in the AD and MD values of CB_R in specific regions, suggesting that increased AD and MD of CB_R may represent early-phase abnormalities in ADHD. Our conjecture is that CB_R plays a pivotal role in the initial stages of ADHD, particularly in its anterior and posterior components. By means of AFQ analyses, more precise localization data can be ascertained regarding damaged fiber segments.

Furthermore, our study revealed no statistically significant alterations in FA along fiber tracts. Our findings are consistent with previous research, which has demonstrated that there are no significant differences in FA within the white matter structure of the cingulum tract between individuals with ADHD and healthy controls (65–68). However, prior investigations have produced inconsistent results, with some diffusion studies indicating reduced FA in the right cingulum bundle among individuals with ADHD in comparison to healthy controls (69, 70). The inconsistency of studies may be attributed to the ADHD subtype or study age range, as well as the potential influence of varying research methods and subject ages. Amlien’s review demonstrated that an increase in AD led to a reduction in FA differences between groups (71). It is plausible that a significant increase in AD may have impacted the FA results in our study, necessitating a larger sample size and replication of the study.

The results of our study indicate that the altered white matter parameters observed in individuals with ADHD were not found to be significantly correlated with clinical test scores. However, the ADHD group exhibited significantly higher scores in attention deficit and hyperactivity, as well as in the areas of inhibition, shifting, emotional control, initiation, working memory, planning, organization of materials, and monitoring, thereby confirming the presence of executive function deficits in this population.

An AFQ analysis of adults with ADHD found that there were no differences between ADHD and HC, and the ADHD group showed no significant association (72), this correlation result is consistent with our findings. Future research is needed to explore the relationship between changes in white matter microstructure and executive function.

### 4.1. Limitations

To the best of our knowledge, this investigation represents a pioneering effort in scrutinizing the precise locations of individual fiber tracts in children with ADHD utilizing the AFQ. Nonetheless, it is imperative to acknowledge certain constraints of this study despite its merits. Firstly, our prospective inquiry was executed on a modest sample size, and additional research is necessary on a more comprehensive sample. Secondly, there was no categorization for ADHD, and future investigations will necessitate a thorough analysis of diverse subtypes of data. Thirdly, the present study was limited by the absence of longitudinal research as all patients were diagnosed during their initial visit. Future studies should investigate the impact of disease progression on white matter microstructure. Fourth, certain fibers, including CH_L, CH_R, and AF_R, were not trackable due to threshold settings in fiber tracking. Fifth, the correlation analyses indicated that the altered white matter parameters did not exhibit a significant correlation with clinical test scores in ADHD, possibly due to the small sample size and the effect of mixing multiple subtypes.

## 5. Conclusion

According to the AFQ method, capable of detecting mild changes in white matter, we identified white matter abnormalities in fiber tracts with a greater degree of spatial precision. As a surveillance tool for ADHD, the AFQ method can detect ADHD-related white matter abnormalities with greater sensitivity, and the CB_R played a critical role in furthering our understanding of ADHD-related white matter abnormalities. Our findings may be helpful in further studying the relationship between focal white matter abnormalities and ADHD.

## Data availability statement

The raw data supporting the conclusions of this article will be made available by the authors, without undue reservation.

## Ethics statement

The studies involving humans were approved by Taihe Hospital, Hubei University of Medicine, Shiyan, Hubei. The studies were conducted in accordance with the local legislation and institutional requirements. Written informed consent for participation in this study was provided by the participants’ legal guardians/next of kin.

## Author contributions

RH and FT: study design, data collection and curation, image scanning and post-processing, formal analysis, visualization, and writing-original draft. WC, YW, YJ, WD, YZ, and BG: case collection, investigation, data curation, image post-processing, software application, formal analysis. QS: scanning specification, technical guidance, supervision. YM: conceptualization, study design, funding acquisition, supervision, and writing – review and editing. All authors contributed to the article and approved the submitted version.

## Conflict of interest

The authors declare that the research was conducted in the absence of any commercial or financial relationships that could be construed as a potential conflict of interest.

## Publisher’s note

All claims expressed in this article are solely those of the authors and do not necessarily represent those of their affiliated organizations, or those of the publisher, the editors and the reviewers. Any product that may be evaluated in this article, or claim that may be made by its manufacturer, is not guaranteed or endorsed by the publisher.

## Footnotes

^1^ https://www.nitrc.org/projects/mricron

^2^ https://fsl.fmrib.ox.ac.uk/fsl/

^3^ https://fsl.fmrib.ox.ac.uk/fsl/fslwiki/Randomise/UserGuide

^4^ https://github.com/yeatmanlab/AFQ
