# Supplementary material for: Microstructure abnormalities of the diffusion quantities in children with attention-deficit/hyperactivity disorder: an AFQ and TBSS study
Source: Front Psychiatry. 2023 Aug 22;14:1237113. doi: 10.3389/fpsyt.2023.1237113 (PMC10477457; doi:10.3389/fpsyt.2023.1237113)
Supplement: Supplementary file 1 [file Table_1.DOCX]

**Supplementary Materials**

**Supplementary Table1 as a real table of clinical characteristics for HC and ADHD groups**

| Category | Gender | Age | Education years | Attention defect score | Hyperactivity score | Inhibit | Shift | Emotional control | Initiate | Working Memory | Plan | Organization of materials | Monitor |
| --- | --- | --- | --- | --- | --- | --- | --- | --- | --- | --- | --- | --- | --- |
| ADHD | M | 9 | 6 | 2.6 | 2.3 | 62 | 64 | 58 | 53 | 54 | 56 | 52 | 59 |
| ADHD | F | 10 | 7 | 2.2 | 1.56 | 52 | 56 | 69 | 49 | 70 | 65 | 54 | 78 |
| ADHD | F | 11 | 8 | 1.89 | 1.11 | 55 | 52 | 49 | 66 | 69 | 47 | 61 | 67 |
| ADHD | M | 13 | 10 | 2 | 1.33 | 63 | 52 | 48 | 70 | 62 | 72 | 63 | 69 |
| ADHD | F | 9 | 6 | 2.7 | 2.2 | 60 | 59 | 58 | 43 | 66 | 75 | 63 | 81 |
| ADHD | M | 9 | 6 | 1.56 | 1.78 | 66 | 47 | 51 | 59 | 56 | 65 | 67 | 78 |
| ADHD | F | 8 | 4 | 1.67 | 1.67 | 52 | 44 | 51 | 52 | 48 | 47 | 51 | 69 |
| ADHD | F | 10 | 7 | 1.33 | 1.78 | 57 | 53 | 58 | 63 | 58 | 58 | 49 | 53 |
| ADHD | M | 10 | 7 | 3 | 2.3 | 49 | 50 | 47 | 41 | 47 | 50 | 55 | 47 |
| ADHD | M | 8 | 5 | 2.2 | 1.1 | 51 | 60 | 54 | 69 | 69 | 54 | 55 | 56 |
| ADHD | F | 10 | 7 | 2.3 | 1 | 52 | 53 | 49 | 59 | 70 | 65 | 57 | 65 |
| ADHD | F | 9 | 6 | 1.78 | 1.11 | 40 | 41 | 40 | 40 | 44 | 45 | 34 | 38 |
| ADHD | M | 7 | 4 | 1.6 | 0.7 | 36 | 37 | 35 | 36 | 45 | 52 | 46 | 38 |
| ADHD | M | 11 | 8 | 1.78 | 1.78 | 68 | 45 | 37 | 53 | 60 | 58 | 66 | 69 |
| ADHD | M | 9 | 6 | 2.78 | 2.22 | 60 | 64 | 58 | 59 | 58 | 58 | 49 | 53 |
| ADHD | M | 11 | 8 | 1.8 | 0.8 | 48 | 52 | 51 | 56 | 56 | 54 | 52 | 57 |
| ADHD | M | 8 | 5 | 1.67 | 1.67 | 66 | 50 | 56 | 59 | 63 | 61 | 55 | 66 |
| ADHD | M | 7 | 4 | 2.56 | 1.78 | 49 | 47 | 45 | 65 | 73 | 72 | 53 | 57 |
| ADHD | M | 10 | 7 | 2.22 | 1.89 | 53 | 43 | 49 | 56 | 52 | 44 | 45 | 53 |
| ADHD | M | 6 | 3 | 1.5 | 2.5 | 58 | 47 | 66 | 42 | 48 | 57 | 69 | 60 |
| ADHD | M | 10 | 7 | 1.89 | 1.67 | 44 | 50 | 45 | 42 | 54 | 52 | 61 | 53 |
| ADHD | M | 7 | 4 | 1.89 | 1.67 | 62 | 50 | 66 | 65 | 68 | 72 | 69 | 76 |
| ADHD | M | 6 | 3 | 1.78 | 1.11 | 65 | 47 | 35 | 42 | 60 | 59 | 69 | 76 |
| ADHD | M | 8 | 5 | 2.33 | 1.33 | 46 | 43 | 54 | 47 | 56 | 61 | 61 | 50 |
| ADHD | M | 8 | 5 | 2 | 1.33 | 49 | 47 | 45 | 59 | 65 | 61 | 61 | 53 |
| ADHD | M | 7 | 4 | 2 | 1.22 | 47 | 63 | 52 | 61 | 60 | 61 | 50 | 54 |
| ADHD | M | 9 | 6 | 1.56 | 1.89 | 71 | 50 | 65 | 50 | 56 | 63 | 61 | 59 |
| HC | M | 12 | 9 | 1.33 | 0.22 | 40 | 48 | 61 | 59 | 60 | 56 | 43 | 60 |
| HC | M | 11 | 8 | 0.11 | 0.33 | 40 | 38 | 37 | 38 | 38 | 44 | 34 | 39 |
| HC | M | 13 | 10 | 0.44 | 0 | 40 | 38 | 40 | 35 | 40 | 42 | 43 | 45 |
| HC | F | 9 | 6 | 0.33 | 0.33 | 40 | 44 | 38 | 46 | 46 | 45 | 37 | 44 |
| HC | F | 10 | 7 | 0.67 | 0.22 | 40 | 44 | 40 | 40 | 41 | 42 | 37 | 38 |
| HC | F | 7 | 4 | 0.66 | 0.78 | 46 | 41 | 50 | 43 | 47 | 54 | 42 | 40 |
| HC | F | 12 | 9 | 0.44 | 0.11 | 42 | 45 | 37 | 40 | 43 | 43 | 43 | 46 |
| HC | M | 10 | 7 | 1.4 | 0.77 | 55 | 43 | 56 | 75 | 65 | 71 | 61 | 66 |
| HC | F | 6 | 3 | 0.55 | 0.33 | 41 | 64 | 55 | 63 | 52 | 51 | 39 | 40 |
| HC | F | 12 | 9 | 0.89 | 0.89 | 49 | 52 | 51 | 46 | 50 | 53 | 64 | 61 |
| HC | M | 13 | 10 | 0.89 | 0.78 | 53 | 38 | 56 | 44 | 47 | 47 | 52 | 45 |
| HC | F | 8 | 5 | 1 | 0 | 42 | 47 | 38 | 43 | 50 | 42 | 34 | 35 |
| HC | F | 9 | 6 | 1.33 | 0.22 | 52 | 47 | 47 | 59 | 52 | 55 | 69 | 49 |
| HC | M | 12 | 9 | 0.44 | 0.11 | 43 | 48 | 42 | 41 | 45 | 40 | 34 | 39 |
| HC | F | 10 | 7 | 1.22 | 0.44 | 40 | 44 | 36 | 40 | 46 | 45 | 51 | 41 |
| HC | M | 7 | 4 | 0.78 | 0.78 | 42 | 37 | 35 | 49 | 48 | 43 | 56 | 54 |
| HC | M | 7 | 4 | 0.78 | 0.56 | 38 | 37 | 35 | 36 | 40 | 43 | 37 | 38 |
| HC | F | 11 | 8 | 0.11 | 0 | 42 | 42 | 37 | 36 | 40 | 43 | 46 | 46 |
| HC | M | 10 | 7 | 0.78 | 0.33 | 49 | 64 | 45 | 59 | 38 | 46 | 45 | 47 |
| HC | M | 7 | 4 | 1.3 | 1.2 | 58 | 37 | 47 | 49 | 53 | 54 | 50 | 57 |
| HC | F | 9 | 6 | 1.11 | 0.56 | 47 | 44 | 51 | 46 | 59 | 47 | 45 | 52 |
| HC | M | 11 | 8 | 0.33 | 0 | 40 | 41 | 48 | 35 | 38 | 37 | 34 | 36 |
